# Supplementary material for: Conformational and Structural Changes in Chickpea Proteins Caused by Simulated Salivary Alterations in the Elderly
Source: Foods. 2023 Oct 5;12(19):3668. doi: 10.3390/foods12193668 (PMC10572786; doi:10.3390/foods12193668)
Supplement: Supplementary file 1 [file foods-12-03668-s001.zip › foods-2625433-supplementary.pdf]

Supplementary material

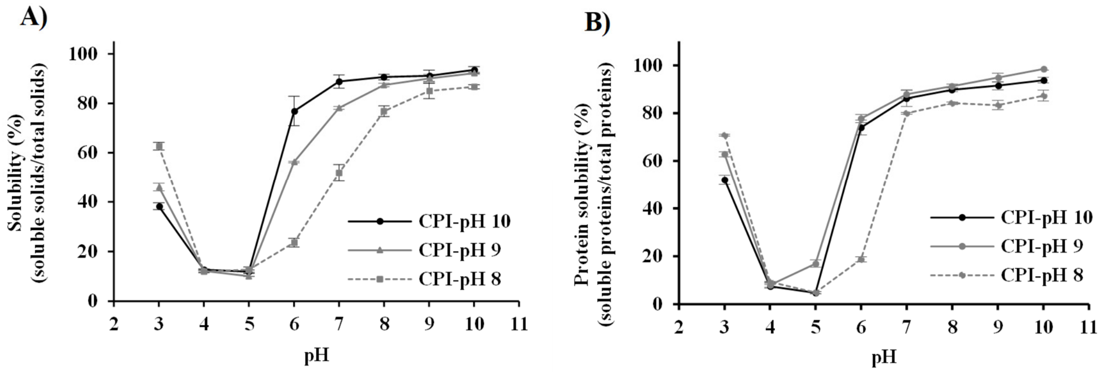

Figure S1. (**A**) *Solubility* (%, g soluble solids/g total solids x100) and (**B**) *protein solubility* (%, g soluble proteins/g total proteins x100) as a function of pH for CPI obtained at three different alkaline extraction pH values (8.0, 9.0, and 10.0). Data are shown as mean ± SD (n=3).

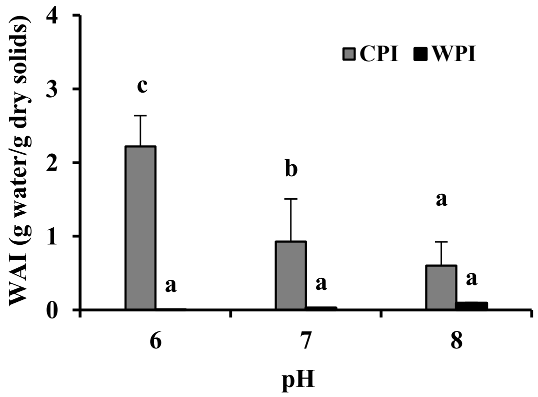

Figure S2. Water absorption index (WAI) (g water/g dry solids) over three pH of simulated salivary fluid values (6, 7, and 8) for CPI and WPI at 37°C. Data are shown as the mean ± SD (n=3). Different letters on each bar of the same isolate indicate significant differences ($p<0.05$).

Table S1. Composition of simulated oral fluid made up to 400 mL and adult (-A) and elderly (-EL) conditions for *in vitro* oral processing.

Compound	Stock solutions (g/L)	Volume to add from stock solution (ml)	
		Adult (-A)	Elderly (-EL)
**Electrolytes:**			
KCl	37.3	15.1	30.2
KH_2PO_4	68	3.7	3.7
$NaHCO_3$	84	6.8	6.8
NaCl	117	0.6	0.6
$MgCl_2(H2O)_6$	30.5	0.5	0.5
$(NH_4)_2CO_3$	48	0.06	0.06
**Conditions for simulated oral processing:**			

$CaCl_2(H_2O)_2$	44.1	0.025	0.025
α-amylase [U/mL]		75	150
pH		7	8

Table S2. Proximate analysis and amino acid content (g/100 g protein) of chickpea protein isolate (CPI) and whey protein isolate (WPI) (%, dry basis)

**Parameters**	**CPI**	**WPI**
Protein (Nx6.25)	88.5	92.0
Lipid	2.6	<0.4
Ash	5.6	2.5
NNE	3.0	5.0
Moisture (%, wet basis)	5.0	7.8
Essential amino acids (EA)		
Leucine (Leu)	5.8	8.3
Isoleucine (Ile)	3.1	3.6
Valine (Val)	3.0	3.2
Cysteine (Cys)	2.5	7.6
Methionine (Met)	1.0	1.5
Phenylalanine (Phe)	4.2	2.2
Threonine (Thr)	3.1	4.2
Tyrosine (Tyr)	1.6	2.2
Lysine (Lys)	5.8	6.4
Histidine (His)	1.9	1.5
Total EA (TEA)	*32.1*	*40.9*
Non-essential amino acids (NEA)		
Glycine (Gly)	3.6	1.5
Alanine (Ala)	3.6	5.2
Proline (Pro)	4.7	4.5
Serine (Ser)	5.2	3.5
Arginine (Arg)	8.9	1.4
Aspartic acid (Asp)	10.9	10.0
Glutamic acid (Glu)	16.4	18.4
Total NEA	*53.3*	*44.6*
Total amino acids (TAA)	***85.4***	***85.4***
Ratio TEA: TAA	*0.38*	*0.48*
Ratio Met: Cys	*0.40*	*0.20*
Total acidic AA, %	32.0	33.3
Total basic AA, %	19.5	10.9
Total hydrophobic AA, %	36.9	44.2
Total non-polar AA, %	11.6	11.6

NNE=non-nitrogen extract. Results (mean, n=3)

Based on their polarity and water binding nature the AAs were grouped as acidic (Asp, Glu), basic (Lys, His, and Arg), hydrophobic (Leu, Ile, Val, Cys, Met, Phe, Gly, Ala, and Pro), and uncharged polar (Thr, Tyr, and Ser).
